# Baicalein Ameliorates Insulin Resistance of HFD/STZ Mice Through Activating PI3K/AKT Signal Pathway of Liver and Skeletal Muscle in a GLP-1R-Dependent Manner

**DOI:** 10.3390/antiox13101246

**Published:** 2024-10-16

**Authors:** Na Liu, Xin Cui, Tingli Guo, Xiaotong Wei, Yuzhuo Sun, Jieyun Liu, Yangyang Zhang, Weina Ma, Wenhui Yan, Lina Chen

**Affiliations:** 1 Department of Pharmacology, School of Basic Medical Sciences, Xi’an Jiaotong University, Xi’an 710061, China; liluna588125@stu.xjtu.edu.cn (N.L.); XL0101@stu.xjtu.edu.cn (X.C.); guotingli@stu.xjtu.edu.cn (T.G.); 3120115055@stu.xjtu.edu.cn (X.W.); sunyuzhuo@stu.xjtu.edu.cn (Y.S.); 3122315002@stu.xjtu.edu.cn (J.L.); Zhangyangyang@stu.xjtu.edu.cn (Y.Z.); 2 School of Pharmacy, Xi’an Jiaotong University, Xi’an 710049, China; maweina2015@xjtu.edu.cn; 3 Key Laboratory of Environment and Genes Related to Diseases (Xi’an Jiaotong University), Ministry of Education, Xi’an 710061, China; 4 Cardiometabolic Innovation Center, Ministry of Education, Xi’an 710061, China; 5 Department of Endocrinology and Second Department of Geriatrics, The First Affiliated Hospital of Xi’an Jiaotong University, Xi’an 710061, China

**Keywords:** baicalein, GLP-1R, insulin resistance, PI3K-AKT, Ca^2+^

## Abstract

Insulin resistance (IR) is the principal pathophysiological change occurring in diabetes mellitus (DM). Baicalein, a bioactive flavonoid primarily extracted from the medicinal plant *Scutellaria baicalensis Georgi*, has been shown in our previous research to be a potential natural glucagon-like peptide-1 receptor (GLP-1R) agonist. However, the exact therapeutic effect of baicalein on DM and its underlying mechanisms remain elusive. In this study, we investigated the therapeutic effects of baicalein on diabetes and sought to clarify its underlying molecular mechanisms. Our results demonstrated that baicalein improves hyperglycemic, hyperinsulinemic, and glucometabolic disorders in mice with induced diabetes via GLP-1R. This was confirmed by the finding that baicalein’s effects on improving IR were largely diminished in mice with whole-body *Glp1r* ablation. Complementarily, network pharmacology analysis highlighted the pivotal involvement of the phosphatidylinositol-3-kinase (PI3K)/protein kinase B (AKT) insulin signaling pathway in the therapeutic actions of baicalein on IR. Our mechanism research significantly confirmed that baicalein mitigates hepatic and muscular IR through the PI3K/AKT signal pathway, both in vitro and in vivo. Furthermore, we demonstrated that baicalein enhances glucose uptake in skeletal muscle cells under IR conditions through the Ca^2+^/calmodulin-dependent protein kinase II (CaMKII)-adenosine 5′-monophosphate-activated protein kinase (AMPK)-glucose transporter 4 (GLUT4) signaling pathway in a GLP-1R-dependent manner. In conclusion, our findings confirm the therapeutic effects of baicalein on IR and reveal that it improves IR in liver and muscle tissues through the PI3K/AKT insulin signaling pathway in a GLP-1R dependent manner. Moreover, we clarified that baicalein enhances the glucose uptake in skeletal muscle tissue through the Ca^2+^/CaMKII-AMPK-GLUT4 signal pathway.

## 1. Introduction

Insulin resistance (IR), defined as a reduced physiological response to insulin in target tissues, is recognized as a pathogenic induction factor for various metabolic syndromes, including nonalcoholic fatty liver disease (NAFLD), atherosclerosis, and type 2 diabetes mellitus (T2DM) [1,2]. Oxidative stress dysregulation is a common pathological mechanism present in these insulin-resistant conditions [3]. Glucagon-like peptide-1 receptor (GLP-1R) agonists, a class of novel hypoglycemic drugs, have exhibited therapeutic effects on multiple metabolic syndromes through diverse mechanisms, including antioxidant, anti-inflammatory, and immunomodulatory actions [4,5,6,7]. Glucagon-like peptide-1 (GLP-1), a native ligand of GLP-1R, modulates postprandial and fasting lipids in hamsters and mice with IR, and activates the central GLP-1R to improve glucose homeostasis in high-fat diet (HFD)-fed mice by increasing insulin levels and enhancing hepatic insulin action [8]. Liraglutide, a GLP-1 analogue, has been shown to improve insulin sensitivity and decrease fasting and postprandial glucose levels prior to weight loss in a GLP-1R-dependent manner [9]. 

The liver, as the foremost site for glucose metabolism, plays a crucial role in regulating the body’s insulin response. Insulin exerts its effects through promoting glycolysis and inhibiting gluconeogenesis, thereby enhancing glucose utilization and reducing glucose production in the body [10]. Skeletal muscle, as the largest organ by mass, is responsible for the overwhelming majority of glucose uptake and storage in physical conditions [11]. There are two major signaling pathways involved in whole-body glucose disposal. The first is the insulin signaling cascade, also known as the insulin-dependent pathway, which is the canonical pathway for glucose metabolism regulation. Key components of the insulin-dependent pathway include the insulin receptor, insulin receptor substrate (IRS), phosphatidylinositol 3-kinase (PI3K), and protein kinase B (AKT) [12], and IR occurs once the net increase in insulin secretion cannot compensate for impaired insulin signaling [13]. The mammalian target of the rapamycin (mTOR)/PI3K/AKT signal pathway is a classic signal transduction pathway for glucose metabolism [14,15]. mTOR activation induces its phosphorylation enhancement, while hyperactivation of mTOR prompts IR and diabetes [16,17]. Glycogen synthase kinase-3β (GSK-3β), belonging to the first kinases and known for phosphorylating glycogen synthase (GS), is inactivated by phosphorylation. Overexpression of GSK-3β is associated with insulin insensitivity, blood glucose regulation disorder, insulin deficiency, and IR [18]. cAMP-responsive element-binding protein (CREB) is defined as a nuclear protein that binds to the cAMP-responsive element (CRE) on the promoter of neuropeptide somatostatin [19]. Overactivation of CREB S133 phosphorylation results in gluconeogenesis increase and glycogenesis decrease in the liver and muscle [20,21]. The other pathway is the insulin-independent pathway, also known as glucose effectiveness, where 5′-adenosine monophosphate-activated protein kinase (AMPK) is a main mediation effector for glucose disposal [22,23]. 

Baicalein (5,6,7-trihydroxyflavone, BAC), a principal bioactive ingredient of *Scutellaria baicalensis Georgi*, has been increasingly recognized for its multiple pharmacological activities, including antibacterial, antiviral, anti-inflammatory, antioxidant and anti-neoplastic activities [24,25,26]. Emerging evidence suggests that multiple factors may contribute to the therapeutic effect of baicalein on diabetes, including α-glucosidase inhibition [27], endoplasmic reticulum stress relief [28], glycation suppression [29], and inflammation remission [30]. However, the explicit mechanisms by which baicalein enhances glucose metabolism remain unclear. In our previous study, baicalein was preliminarily confirmed as a potential GLP-1R agonist in both in vitro and in vivo experiments [31]. In this study, we sought to evaluate the connection between the hypoglycemic effect of baicalein on DM and GLP-1R, and further investigate its underlying mechanisms under IR conditions both in wild-type and *Glp1r* knockout (KO) mice, as well as in HepG2 and C2C12 cells.

## 2. Materials and Methods

### 2.1. Reagents and Materials

Baicalein was purchased from Chengdu Pufei De Biotech Co., Ltd. (Chengdu, China). Exendin (9–39) (≥99%) were purchased from MedChemExpress (City of Kennyworth, NJ, USA). The fetal bovine serum (FBS), Dulbecco’s modified eagle medium (DMEM), modified eagle medium (MEM), and trypsin were obtained from Gibco (GrandIsland, NY, USA). The phosphate buffered saline (PBS), palmitic acid (PA), bovine serum albumin (BSA), penicillin-streptomycin liquid, glycogen content determination kit and bicinchoninic acid (BCA) protein assay kit were supplied from Solarbio Science & Technology Co., Ltd. (Beijing, China). The sodium dodecyl sulfate-polyacrylamide gel electrophoresis (SDS-PAGE) gel kit was purchased from Servicebio (Wuhan, China). Insulin and liraglutide were obtained from Novo Nordisk (Copenhagen, Denmark).

### 2.2. Cell Culture and Treatment

The HepG2 cell line and C2C12 cell line were obtained from Hycyte Biotechnology Co., Ltd. (Shenzhen, China). C2C12 cells were cultured in DMEM medium (complemented with 10% FBS, 100 U/mL penicillin and 100 μg/mL streptomycin) and were differentiated according to procedures previously reported [32]. HepG2 cells were cultured in MEM medium (supplemented with 10% FBS, 100 U/mL penicillin and 100 μg/mL streptomycin). All cells were maintained in a 5% CO_2_, 37 °C cell culture incubator. 

For establishment of insulin-resistant cell models, C2C12 myotubes cells were transferred to DMEM medium containing 250 μM PA for 24 h to construct IR-C2C12 cells [33,34], and HepG2 cells were cultured in MEM medium containing 1 μΜ insulin for 24 h to establish IR- HepG2 cells [33].

### 2.3. Glucose Uptake Assay

HepG2 and C2C12 cells were seeded in 96-well plates (1 × 10^4^ cells/well) and subjected to different drug administrations. After drug intervention, cells were incubated with 2-[N-(7-nitrobenz-2-oxa-1,3-diazol-4-yl) amino]-2-deoxy-D-glucose (2-NBDG, Anjiekai Biological Medicine Technology Co., Ltd., Wuhan, China), a fluorescent glucose analogue, at a final concentration of 100 μM combined with 100 nM insulin for 30 min at 37 °C. Subsequently, the cells were washed 3 times with pre-cold Hank’s solution and the fluorescence intensity was immediately measured by a FlexStation^®^ 3 multi-mode microplate reader (Sunnyvale, CA, USA) at an excitation wavelength of 485 nm and an emission wavelength of 530 nm. An estimation of the overall glucose uptake was obtained by quantifying the fluorescence. The glucose uptake was also visualized using a fluorescence microscope (Nikon, Tokyo, Japan) where Hoechst 33342 nuclear dying buffer (Thermo Fisher Scientific Inc., Waltham, MA, USA) was applied to label the nucleus.

### 2.4. Glucose Consumption Assay

Cellular glucose consumption was assayed by a glucose assay kit (glucose oxidase method) (Nanjing Jiancheng Biological Engineering Research Institute, Nanjing, China). In detail, HepG2 and C2C12 cells were seeded into a 96-well plate at a density of 5 × 10^3^ cells per well with 6 wells left as blanks. After adhesion, the culture medium was replaced with fresh MEM/DMEM medium (free FBS and phenol red) containing 1 μM insulin or 250 μM PA for 24 h. After incubation, the culture medium was removed and cells were given with different administrations for 48 h. At the final 30 min, 100 nM insulin was added to each well and further incubation. Then, the plates were centrifugated (1000× *g*, 25 °C, 10 min), the glucose concentration of the medium was determined following the instrument. The amount of glucose consumption was calculated by subtracting the glucose content in the blank wells from the determination wells.

### 2.5. Ca^2+^ Mobility Determination

C2C12 cells were pre-plated in 96-well plates and cultured to 30% confluence. The culture medium was then removed and subsequently replaced with fresh medium, either alone or combined with 250 μM PA, for 24 h incubation. After treatment, the culture medium was removed and cells were washed twice with pre-warmed calcium imaging buffer (CIB) and stained with buffer containing 2.5 μM Fluo-3 AM Ca^2+^ fluorescent probe (Beyotime Biotechnology, Shanghai, China) and 0.1% F-127 (Sigma-Aldrich, Darmstadt, Germany), and 500 nM exendin (9–39) was arranged to inhibit the GLP-1R in relevant groups. After a 40 min incubation, the dyed cells were washed twice with fresh CIB and immediately imaged under a fluorescence microscope (Nikon, Tokyo, Japan). Different concentrations of analyte solutions (dissolved in CIB) were added to each well at 5 s after initial imaging. Responses were monitored at 3 s intervals for a total of 120 s.

### 2.6. Small Interfering RNA (siRNA) and Transfection

HepG2 and C2C12 cells were pre-seeded in 6-well plates and cultured to 60% confluence in MEM/DMEM medium free of penicillin and streptomycin. *Glp1r* siRNAs were gently mixed with Lip2000 transfection reagent (Solarbio Science & Technology Co., Ltd., Beijing, China) to a final volume of 100 μL mixture and incubated for 20 min at room temperature. The sequence of *Glp1r* siRNAs used was as follows. siRNA1: A: GGCCAGUAGUGUGCUACAATT, AS: UUGUAGCACACUACUACUGGCCTT; siRNA2: A: GCAGCCAACUACUACUGGUTT, AS: ACCAGUAGUAGUUGGCUGCTT; siRNA3: A: GGCUAUCCUGUACUGCUUUTT, AS: AAAGCAGUACAGGAUAGCCTT. Subsequently, the growth medium of culture plates was removed and replaced with 900 μL fresh medium and 100 μL transfection mixture. After incubation for 6 h, the culture medium was removed and further intervention was arranged.

### 2.7. In Vivo Pharmacological Experiment

Animal experiments were conducted to investigate the effects of baicalein on T2DM. HFD (60% fat, 20% protein and 20% carbohydrates) feeding combined with 3 consecutive days of intraperitoneal (i.p.) injection of 60 mg/kg streptozotocin (STZ, Sigma-Aldrich, Darmstadt, Germany) was used to induce T2DM mice models both in male C57BL/6J (WT) mice and male *Glp1r* KO mice [35]. The detailed experiments protocol was as follows. WT mice (*n* = 40, 16–20 g weight, 4–6 weeks old) were purchased from the Medical Experimental Animal Center of Xi’an Jiaotong University. Global *Glp1r* KO mice (*n* = 24) were developed with Cyagen Biosciences Inc. (Suzhou, China). All mice were housed in a temperature-controlled room (22–24 °C) with a 12 h light/dark cycle and had free access to food and water. WT mice were classified into a control group (WT–Control, *n* = 8) and an HFD group (WT–HFD, *n* = 32), and *Glp1r* KO mice were classified into a control group (KO–Control, *n* = 8) and an HFD group (KO–HFD, *n* = 16) randomly according to body weight (BW). Mice in the Control groups were fed with normal chow diets (ND, 10% fat, 20% protein and 70% carbohydrates), while mice in the HFD groups were fed with HFD. Eight weeks later, after fasting for 12 h, mice in the Control groups and HFD groups were i.p. injected with citrate buffer or a corresponding dosage of STZ respectively for 3 consecutive days. Then, after fasting for 12 h, the fasting blood glucose (FBG) of HFD mice was monitored with an automatic blood glucose meter (Sinocare Inc., Changsha, China), and the diabetic mouse model was considered successfully established if the FBG of the mice was over 16.7 mmol/L for two consecutive days. The WT-HFD mice were then randomly divided into 4 groups: Model group (WT-Model, *n* = 8) and Baicalein group (WT-BAC 50, 100, and 200 mg/kg BW, respectively, *n* = 8), and the KO-HFD mice were divided into Model group (KO-Model, *n* = 8) and Baicalein group (KO-BAC 100 mg/kg, *n* = 8). Mice in the Baicalein groups were administered with pre-established dosages of baicalein by gavage (dissolved in 0.5% sodium carboxymethyl cellulose (CMC-Na)) at the same time every day, while mice in Control and Model groups were administered with equivalent 0.5% CMC-Na for 6 weeks. The BW and FBG of each mouse were monitored weekly. At the end of the 6th week, after monitoring FBG, the mice were euthanized and serum was collected for fasting insulin (FINS) monitoring. The liver and skeletal muscle tissues were removed, gently rinsed in cold saline and immediately stored at −80 °C for biochemistry assay.

All animal experimental procedures were approved by the Institutional Animals Care and Use Committee at Xi’an Jiaotong University and were in accordance with the National Institutes of Health Guide for the Care and Use of Laboratory Animals.

### 2.8. Intraperitoneal Glucose Tolerance Test (IPGTT) and Insulin Tolerance Test (ITT)

IPGTT is a conventional protocol used to monitor the peripheral disposal of glucose and insulin secretion over a period of time, and ITT is employed to evaluate whole-body insulin action [35]. During the 6th week, IPGTT and ITT assays were conducted. For IPGTT, mice were fasted for 8 h, and blood glucose levels of below time point were monitored via tail vein blood collection, 0 (prior to glucose injection), 30, 60, and 120 min following the injection of 2 g/kg glucose. For ITT assay, mice underwent an 8 h fasting period before receiving an intraperitoneal injection of 0.75 U/kg insulin, and blood glucose levels were measured at 0 (prior to insulin injection), 15, 30, 60, and 120 min following insulin administration. The area under the curve (AUC) of blood glucose fluctuations was also determined.

### 2.9. RNA Isolation, cDNA Synthesis and Real Time PCR

Total RNA of cells was extracted using RNAiso Plus reagent (Takara, Tokyo, Japan). PrimeScript^TM^ RT reagent kit with gDNA eraser (Takara, Tokyo, Japan) was utilized to purify and reverse-transcribe the RNA into cDNA. The primers used are provided in Table 1. RT-PCR was conducted using TB Green^TM^ Premix Ex Taq^TM^ II (Takara, Tokyo, Japan), and the results were calculated by 2^−ΔΔct^.

### 2.10. Quantifications of Plasm Insulin and Homeostasis Model Assessment Insulin Resistance (HOMA-IR) in Mice

Plasma FINS concentration was quantified using the insulin enzyme-linked immunosorbent assay (ELISA) commercial kit (Cloud-Clone Corp., Wuhan, China) following the manufacturer’s protocols. The HOMA-IR index was calculated using the formula: FBG × FINS/22.5.

### 2.11. Glycogen Content Determination Assay

Approximately 100 mg of liver and skeletal muscle tissues were collected from each mouse and weighed precisely. The tissues were homogenized in 750 μL of alkaline extraction buffer (Solarbio Science & Technology Co., Ltd., Beijing, China) using a Scientz-48 high-throughput tissue grinder (Scientz Biotechnology Co., Ltd., Ningbo, China) and subjected to a boiling water bath for 20 min. After cooling, the volume of the solution was fixed to 4 mL. After mixing and centrifugation, the supernatant was collected, and anthrone and H_2_SO_4_ were added to determine the OD value at 620 nm. Glycogen concentrations in tissues were normalized to tissue weights.

### 2.12. Western Blotting Analysis

Proteins extracted from the liver and skeletal muscle of mice, as well as from cultured HepG2 and C2C12 cells, were used for Western blotting analysis. Briefly, tissues or cells were homogenized in cold radioimmunoprecipitation assay (RIPA) lysis buffer (Solarbio Science & Technology Co., Ltd., Beijing, China). After centrifugation (12,000× *g*, 4 °C, 20 min), the supernatant was collected, and the protein concentration was determined by a BCA kit according to instrument. Approximately 20 μg of proteins were added to a polyacrylamide gel to separate according to molecular weight. The separated proteins were transferred onto polyvinylidene fluoride (PVDF) membranes (Merck Millipore Ltd., Billerica, MA, USA). The blots were blocked with 5% skim milk (*m*/*v*) and incubated with primary antibodies overnight at 4 °C. The primary antibodies used were anti-PGC-1α (1:1000, ABclonal, Wuhan, China), anti-p-mTOR (Ser2481) (1:500, abways Technology, Shanghai, China), anti-mTOR (1:500, abways Technology, Shanghai, China), anti-PI3K-p85 (1:1000, CST, Boston, MA, USA), anti-PI3K (1:1000, Abcam, Cambridge Cambs, UK), anti-p-AKT (Ser473) (1:1000, CST, Boston, MA, USA), anti-AKT (1:1000, Epitomics, Burlingame, CA, USA), anti-p-GSK3β (Ser9) (1:1000, CST, Boston, MA, USA), anti-GSK3β (1:1000, CST, Boston, MA, USA), anti-p-CREB (S133) (1:500, PTM Biolabs., Hangzhou, China), anti-CREB (1:500, PTM Biolabs., Hangzhou, China), anti-GLP-1R (1:500, Proteintech, Wuhan, China), anti-PTP1B (1:2000, Proteintech, Wuhan, China), anti-AMPK (1:1000, Proteintech, Wuhan, China), anti-p-AMPK (Thr172) (1:1000, Proteintech, Wuhan, China), anti-CaMKII (1:500, HuaAn Biotechnology Co., Ltd., Hangzhou, China), anti-p-CaMKII (Thr286) (1:500, HuaAn Biotechnology Co., Ltd., Hangzhou, China), and anti-GAPDH (1:10,000, Proteintech, Wuhan, China). After washing with Tris-HCl buffer solution with Tween-20 (TBST) 3 times, blots were incubated with horseradish peroxidase (HRP)-conjugated secondary antibodies (1:20,000, Proteintech, Wuhan, China) for 2 h, and protein bands were visualized using a chemiluminescence system (Tanon, Shanghai, China).

### 2.13. Immunofluorescence

C2C12 cells were cultured in 24-well plates with circular glass coverslips overnight and given with different administrations. After treatment, the cells were washed with pre-cold PBS 3 times, fixed with 4% paraformaldehyde, blocked with 5% (*m*/*v*) BSA and incubated overnight with GLUT4 antibody (1:100, ABclonal, Wuhan, China). After washing with PBS 3 times, the cells were incubated with a fluorophore-conjugated secondary antibody (1:500, Bioscience Biotechnology Co., Ltd., Shanghai, China) for 2 h at room temperature. Fluorescence-inverted microscopy (Leica, Wetzlar, Germany) and two-photon fluorescence microscopy (Leica, Wetzlar, Germany) were used for imaging records.

### 2.14. Network Pharmacology

#### 2.14.1. Gene Collection of Insulin Resistance

Genes related to insulin resistance were gathered from the DisGeNET, GeneCards and NCBI databases using “Insulin Resistance” as the search term.

#### 2.14.2. Prediction and Identification of Potential Targets of Baicalein

The Traditional Chinese Medicine Systems Pharmacology (TCMSP) database, PUBCHEM database and SwissTargetPrediction database were utilized to predict the associated targets of baicalein. 

The overlapping targets from the two datasets were analyzed to identify the potential target set of baicalein on treating insulin resistance, and the target information was validated using the UniProt database (https://www.uniprot.org/ (accessed on 1 December 2023)). Cytoscape software (Version: 3.7.1) (https://cytoscape.org/ (accessed on 16 December 2023)) was applied to construct the component-target network.

#### 2.14.3. Gene Ontology (GO) Functional Enrichment and Kyoto Encyclopedia of Genes and Genomes (KEGG) Pathway Analysis

ClusterProfiler (Version: 4.2.1) was used to perform GO functional annotation and KEGG pathway enrichment analysis on the target set of baicalein in the treatment of insulin resistance. GO functional annotation and KEGG pathway enrichment analysis were performed using gene annotation and signal pathway directories respectively, and *p* value < 0.05 and q value < 0.05 were considered as the significance criteria.

#### 2.14.4. Key Target Discovery Based on Protein–Protein Interaction (PPI) Network Analysis

Important targets within the PPI network are known to play pivotal roles in diseases. Therefore, the PPI network was analyzed from a network module perspective to identify key targets involved in the therapeutic effect of baicalein on insulin resistance. Network topology parameters such as degree, betweenness, and closeness of targets were analyzed using the CytoNCA tools within the Cytoscape software (Version: 3.7.1). This analysis aimed to identify significant nodes within the network and focused on maximum group mining of network modules.

### 2.15. Statistical Analyses

The results were presented as mean ± standard error of mean (SEM). Statistical comparisons among multiple groups were performed using one-way ANOVA followed by the least significant difference (LSD) post hoc test. A value of *p* < 0.05 was regarded as statistically significant.

## 3. Results

### 3.1. Baicalein Ameliorated Hyperglycemia of T2DM Mice by Alleviating IR

**Figure 1:**
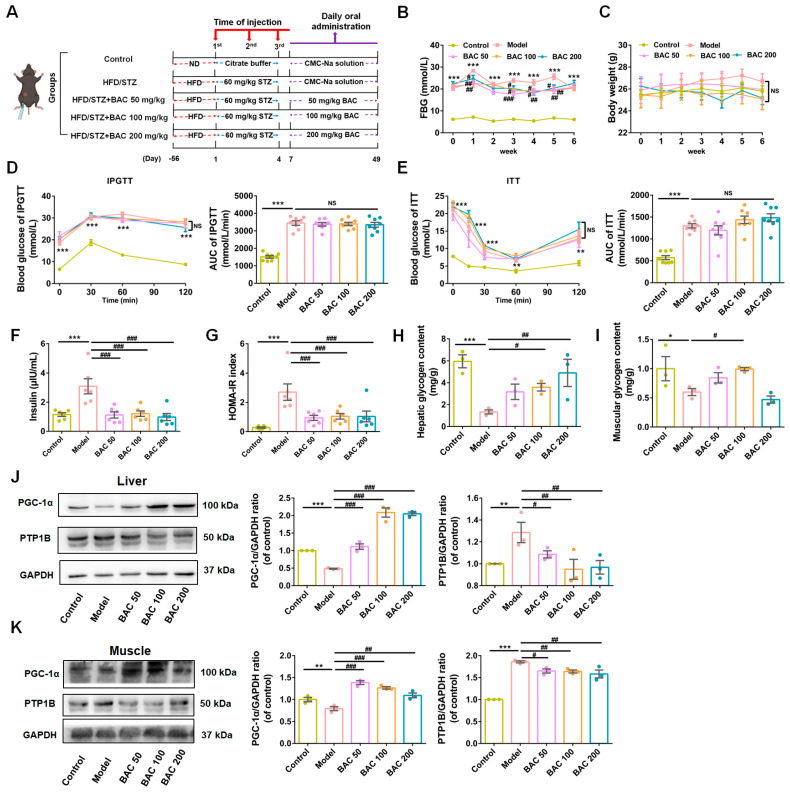
Baicalein improved hyperglycemia of T2DM mice mainly through alleviating insulin resistance (IR) occurring in liver and skeletal muscle tissues. (**A**) A scheme of experimental grouping in wild-type (WT) mice. (**B**) The fasting blood glucose (FBG) level (*n* = 8), (**C**) body weight (*n* = 8), (**D**) IPGTT (*n* = 8), (**E**) ITT (*n* = 8), (**F**) fasting blood insulin (*n* = 6), and (**G**) HOMA-IR (*n* = 6) determination performed in WT mice. (**H**,**I**) Measurement of glycogen content in liver (**H**) and skeletal muscle tissues (**I**) (*n* = 3). (**J**,**K**) Representative immunoblots (left) and quantification results (right) of PGC-1α and PTP1B in liver (**J**) and skeletal muscle (**K**) of WT mice given different administrations (*n* = 3). Data were presented as mean ± SEM. * *p* < 0.05, ** *p* < 0.01, *** *p* < 0.001 of control; ^#^ *p* < 0.05, ^##^ *p* < 0.01, ^###^ *p* < 0.001 of model, one-way ANOVA. NS: no significance.

As depicted in Figure 1A, the mice in the HFD/STZ groups were fed HFD combined with i.p. injections of STZ, and baicalein was administrated orally daily to investigate its therapeutic effects on diabetes, while the mice in the Control group were fed with ND and injected with isodose citrate buffer. As shown in Figure 1B,C, the FBG of mice in the Model group was significantly higher than that in the Control group, and compared to the Model group, baicalein administration significantly ameliorated the high blood glucose levels of T2DM mice, while the group BAC 100 mg/kg had more steady and effective hypoglycemic effect than the other two dosage groups (Figure 1B). There was no significant discrepancy on BW among the groups (Figure 1C). Additionally, T2DM mice exhibited an impaired glucose tolerance and insulin sensitivity in IPGTT and ITT compared to their littermates in the Control group, while baicalein administration exerted minimal influence on these parameters (Figure 1D,E). These confounding results prompted us to further investigate the hypoglycemic mechanism of baicalein on diabetes. Our ongoing investigation revealed that, compared to the Control group, the mice in the T2DM group had higher plasma insulin levels and a higher HOMA-IR ratio, and baicalein administration ameliorated the hyperinsulinemia and insulin homeostasis imbalance occurring in T2DM mice (Figure 1F,G). These results prompted us to consider whether baicalein improves glucose metabolism of T2DM mice through ameliorating IR. Considering liver and skeletal muscle tissues are primary sites for glucose disposal, we assessed glycogen content in these tissues. The results indicated that, compared with the Control group, the mice in the Model group presented significantly decreased glycogen content both in liver and muscle, and the administration of baicalein compensated for this trend (Figure 1H,I). Further experiments were arranged to determine the IR-related proteins, including protein tyrosine phosphatase 1B (PTP1B) and peroxisome proliferator-activated receptor-γ coactivator (PGC)-1α [36,37]. As shown in Figure 1J,K, compared to the Control group, the protein level of PTP1B was upregulated, while PGC-1α was downregulated in the Model group, and the administration of baicalein increased the expression of PGC-1α and decreased the level of PTP1B in T2DM mice both in liver and skeletal muscle tissues. Based on the information acquired above, it was reasonable for us to consider that baicalein improves glucose metabolism of T2DM mice through improving IR occurring in liver and muscle tissues. 

### 3.2. Network Pharmacology Analysis Applied to Clarify the Potential Signal Pathway Involved in the IR Improvement Effect of Baicalein

**Figure 2:**
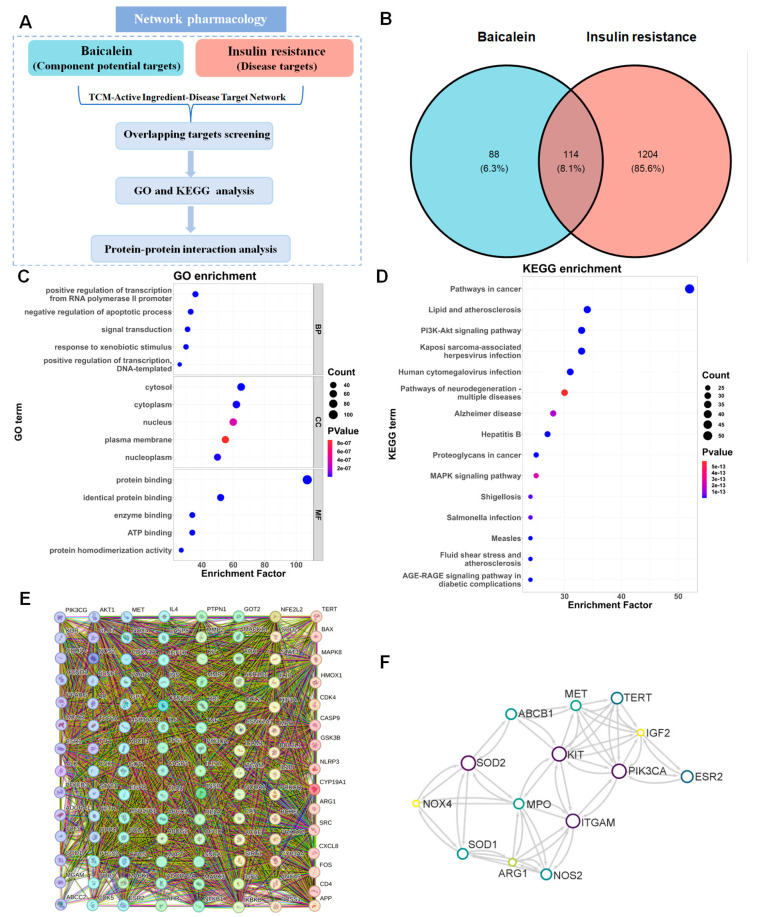
The network pharmacological analysis results of baicalein and insulin resistance (IR). (**A**) A scheme of network pharmacological analysis assay. (**B**) Venn diagram of baicalein and IR. (**C**) Analysis of Gene Ontology (GO) pathway enrichment. (**D**) Analysis of Kyoto Encyclopedia of Genes and Genomes (KEGG) pathway enrichment. (**E**) Protein–protein interaction (PPI) network of 114 crosslinking targets of baicalein and IR. (**F**) The simplified PPI network of crucial targets of baicalein and IR.

Network pharmacology analysis was employed to elucidate the potential signal pathway involved in the improvement effect of baicalein on IR. The detailed protocol is depicted in Figure 2A.

#### 3.2.1. Prediction of the Target Proteins

The keyword “baicalein” was used for target identification, and we recognized 202 baicalein-related targets from TCMSP, PUBCHEM, and Swisstarget databases. “Insulin resistance” was searched as keyword for disease-related targets, and 1318 IR-related targets were obtained from DisGenNet, GeneCards, and NCBI databases. A total of 114 overlapping targets between “baicalein” and “insulin resistance” were selected as potential targets for baicalein against IR (Figure 2B).

#### 3.2.2. GO and KEGG Enrichment Analyses

The potential targets were imported to implement GO enrichment analysis, where biological process (BP), cellular component (CC), and molecular function (MF) were the top 3 terms. As illustrated in Figure 2C, BP-related items primarily involved the positive regulation of transcription from the RNA polymerase II promoter, negative regulation of the apoptotic process, and signal transduction. Cytosol, cytoplasm, and nucleus were contained in CC items. MF-related items mainly included protein binding, identical protein binding, and enzyme binding.

The ClusterProfiler program (Version: 4.2.1) was applied to implement the KEGG enrichment analysis. And ggplot2 programming package (Version: 3.5.1) was used to visualize the results.The results indicated that 157 probable pathways might participate in this process, and the top 3 pathways involved were pathways in cancer, lipid and atherosclerosis, and the PI3K-AKT signaling pathway (Figure 2D and Table S1).

#### 3.2.3. PPI Network

The 114 potential targets were imported into the Cytoscape software to construct the PPI network. Notably, PIK3CG (PI3Kγ/p110γ, a key regulator molecule in the pathological processes of inflammation and oxidation), PIK3CA (a gene encoding the p110α catalytic subunit of PI3K), and Mitogen-activated protein kinases (MAPK) 1 and 14 were identified as interacting with other targets in the PPI network (Figure 2E). The hub genes were then screened by CytoHubba. The top 14 genes, including PIK3CA, estrogen receptor 2 (ESR2), insulin-like growth factor 2 (IGF2), telomerase reverse transcriptase (TERT), hepatocyte growth factor receptor (MET), KIT (a tyrosine kinase receptor belonging to subclass III of receptor tyrosine kinases), ABCB1 (a gene that encodes P-glycoprotein), superoxide dismutase 1 (SOD1), superoxide dismutase 2 (SOD2), myeloperoxidase (MPO), nitric oxide synthase (NOS2), arginase 1 (ARG1), NADPH oxidase 4 (NOX4), and integrin subunit alpha M (ITGAM) were identified as hub genes involved in the beneficial effects of baicalein on IR (Figure 2F).

Taken all together, the results of network pharmacology analysis revealed that the PI3K-AKT signaling pathway is potentially involved in the beneficial effects of baicalein against IR. 

### 3.3. Baicalein Improved Glucose Disposal of Liver and Skeletal Muscle Tissues Through PI3K/AKT Insulin Signaling Pathway

**Figure 3:**
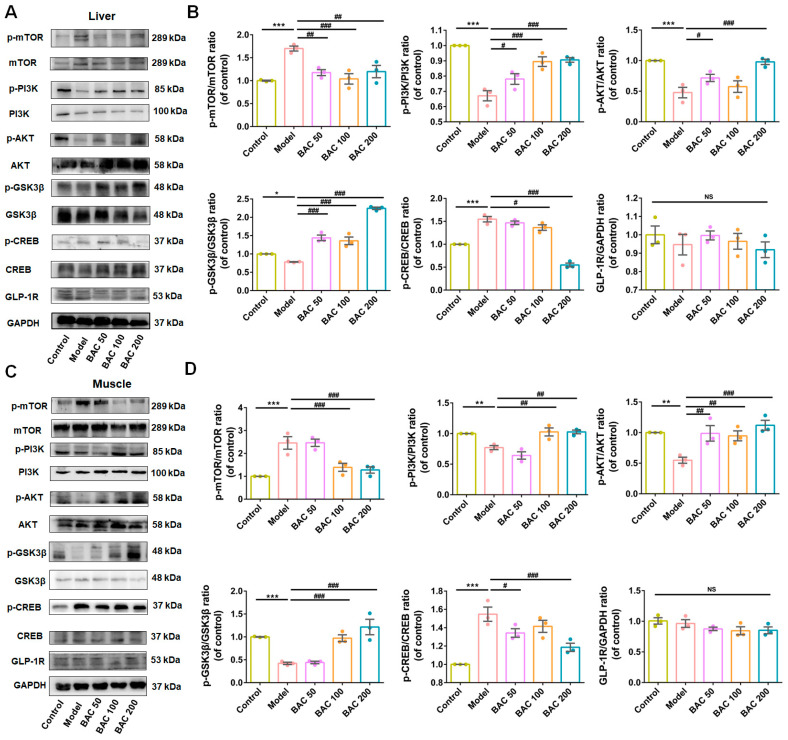
Baicalein ameliorated insulin resistance occurring in liver and skeletal muscle tissues in T2DM mice through the PI3K/AKT signaling pathway. (**A**) Representative Western blot bands of p-mTOR, mTOR, p-PI3K, PI3K, p-AKT, AKT, p-GSK3β, GSK3β, p-CREB, CREB, and GLP-1R in liver tissue of WT mice. (**B**) The quantification results of p-mTOR/mTOR, p-PI3K/PI3K, p-AKT/AKT, p-GSK3β/GSK3β, p-CREB/CREB, and GLP-1R/GAPDH ratios (*n* = 3). Densitometry values of the Western blotting were normalized and represented as relative intensity. (**C**) Representative Western blot bands for p-mTOR, mTOR, p-PI3K, PI3K, p-AKT, AKT, p-GSK3β, GSK3β, p-CREB, CREB, and GLP-1R in skeletal muscle tissue of T2DM mice. (**D**) The quantification results of above proteins expression in skeletal muscle tissue (*n* = 3). Data were expressed as mean ± SEM, * *p* < 0.05, ** *p* < 0.01, *** *p* < 0.001 of control; ^#^ *p* < 0.05, ^##^ *p* < 0.01, ^###^ *p* < 0.001 of model, one-way ANOVA. NS: no significance.

Liver and skeletal muscle are major insulin-target tissues that play crucial roles in whole-body blood glucose disposal [38], where insulin-stimulated glucose utilization impairment is significantly correlated with IR [39]. Proteins related to the PI3K/AKT signaling pathway were determined to verify our hypothesis. As depicted in Figure 3, compared to the Control group, the levels of p-PI3K/PI3K, p-AKT/AKT, and p-GSK3β/GSK3β in the Model group were decreased, while the levels of p-mTOR/mTOR and p-CREB/CREB were increased both in liver and muscle. With the administration of baicalein, the levels of these proteins returned to normal condition. Collectively, these findings demonstrated that baicalein improves glucose metabolism through enhancing glucose disposal in liver and skeletal tissues through the activation of the PI3K/AKT signaling pathway. 

### 3.4. Glp1r KO Disrupted the Beneficial Effects of Baicalein on Glucose Metabolism

**Figure 4:**
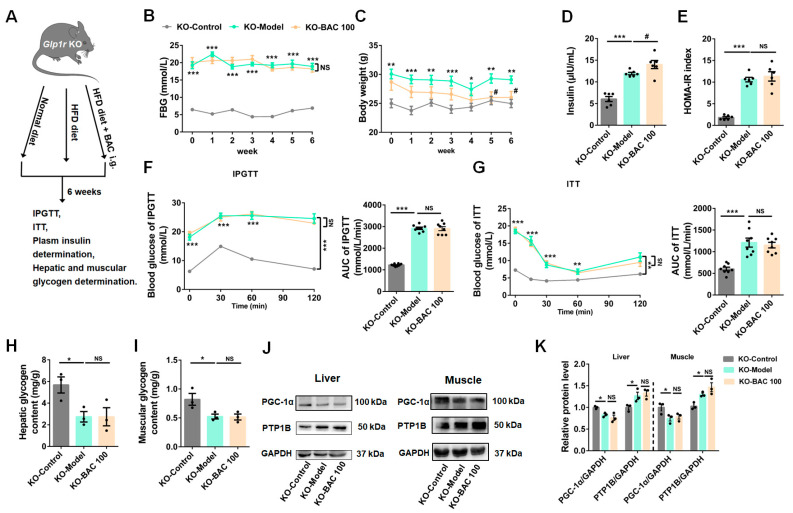
Glucagon-like peptide-1 receptor (*Glp1r*) knockout (KO) counteracted the therapeutic effect of baicalein on diabetes-induced mice. (**A**) A scheme of experimental arrangement in *Glp1r* KO mice. (**B**–**G**) The results of baicalein administration on FBG (*n* = 8) (**B**), body weight (*n* = 8) (**C**), fasting blood insulin (*n* = 6) (**D**), HOMA-IR (*n* = 6) (**E**), IPGTT (*n* = 8) (**F**), and ITT (*n* = 8) (**G**) in *Glp1r* KO mice. (**H**,**I**) The glycogen content determination in liver (**H**) and skeletal muscle (**I**) of *Glp1r* KO mice (*n* = 3). (**J**) Representative Western blot bands for PGC-1α and PTP1B expression in liver and muscle tissues. (**K**) Quantification results of Western blots in liver and muscle (*n* = 3). Data were presented as mean ± SEM, * *p* < 0.05, ** *p* < 0.01, *** *p* < 0.001 of KO-Control, ^#^ *p* < 0.05 of KO-Model, one-way ANOVA. NS: no significance.

**Figure 5:**
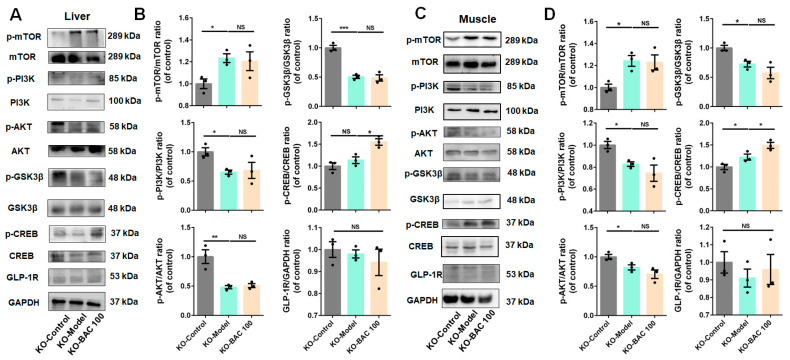
The invalidated effects of baicalein on glucose metabolism among *Glp1r* KO mice were related to disabled PI3K/AKT signaling pathways in liver and skeletal muscle. (**A**) Representative Western blot bands for p-mTOR, mTOR, p-PI3K, PI3K, p-AKT, AKT, p-GSK3β, GSK3β, p-CREB, CREB, and GLP-1R in liver tissue. (**B**) The quantification results of above proteins in liver tissue (*n* = 3). (**C**) Representative Western blot bands for p-mTOR, mTOR, p-PI3K, PI3K, p-AKT, AKT, p-GSK3β, GSK3β, p-CREB, CREB, and GLP-1R in skeletal muscle of *Glp1r* KO mice given different administrations. (**D**) The quantification results of above proteins in skeletal muscle (*n* = 3). Data were presented as mean ± SEM, * *p* < 0.05, ** *p* < 0.01, *** *p* < 0.001. NS: no significance.

Experiments were implemented in *Glp1r* KO mice to elucidate the connection between baicalein’s effect on blood glucose regulation and GLP-1R. As shown in Figure 4A, *Glp1r* KO mice were randomly divided into 3 groups and given treatment similar to their WT counterparts, and 100 mg/kg BW baicalein was chosen as the therapeutic dosage according to its optimal therapeutic effect in WT mice. Our results indicated that, compared to mice in the KO-Control group, the mice in the KO-Model group exhibited higher FBG, BW, FINS, HOMA-IR, and worse reactivity to glucose and insulin in IPGTT and ITT assay. However, compared to the KO-Model group, baicalein administration had minimal effects on FBG, FINS, HOMA-IR, IPGTT, and ITT, although it had a modest recovery effect on overweight (Figure 4B–G). Additionally, under *Glp1r* KO conditions, HFD/STZ administration induced decreased hepatic and muscular glycogen content, while baicalein administration had minimal effect on reversing the abnormal decrease in hepatic and muscular glycogen content in the Model group (Figure 4H,I). Moreover, similar to the phenomenon observed in WT mice, compared to the KO-Control group, the protein level of PGC-1α was downregulated, and the protein level of PTP1B was upregulated in the KO-Model group, while baicalein treatment had minimal recovery effect under *Glp1r* KO conditions (Figure 4J,K). Our mechanism investigation indicated that with *Glp1r* KO, the activation effects of baicalein on the PI3K/AKT insulin signaling pathway were abolished both in liver and muscle tissues (Figure 5A–D). Overall, our data demonstrated that the glucometabolic improvement effect of baicalein on diabetes was largely attenuated with *Glp1r* KO. 

### 3.5. GLP-1R Was Indispensable for Baicalein to Improve Glucose Utilization in IR-HepG2 and IR-C2C12 Cells

**Figure 6:**
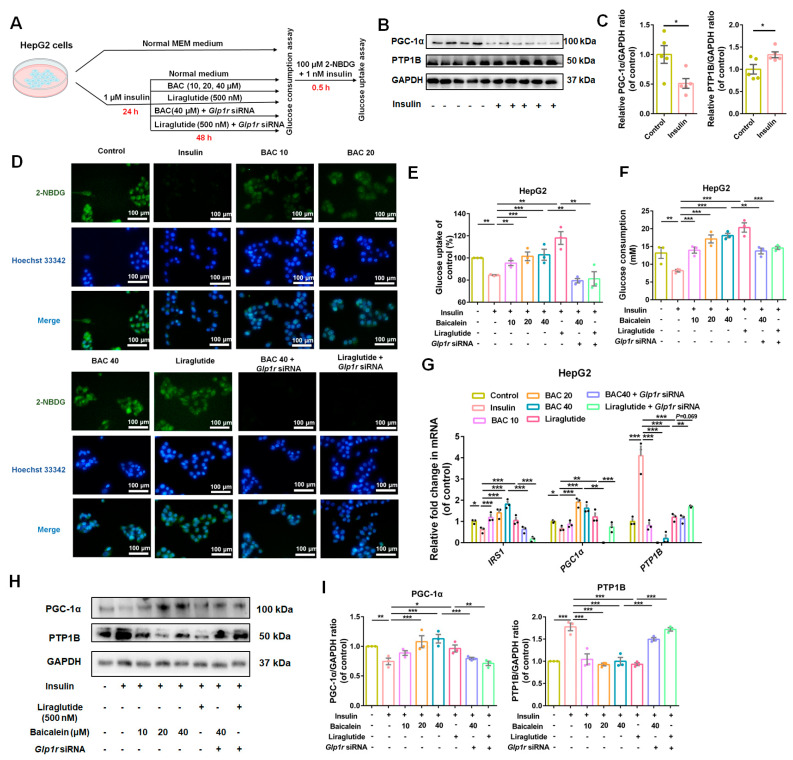
Baicalein enhanced glucose uptake of insulin resistant (IR)-HepG2 cells. (**A**) Investigation schematic for IR model construction and drug administration in HepG2 cells. (**B**) Representative Western blots bands for PGC-1α and PTP1B in HepG2 cells. (**C**) The quantification results of PGC-1α/GAPDH and PTP1B/GAPDH ratios in HepG2 cells (*n* = 5). (**D**) Immunofluorescence staining of 2-NBDG positive cells in control, insulin (1 μM), liraglutide (500 nM), and baicalein (10, 20 and 40 μM) with and without *Glp1r* knockdown. Scale bar: 100 μm. (**E**) Glucose uptake rate of HepG2 cells given with different administrations (*n* = 3). (**F**) Glucose consumption of HepG2 cells given with different treatment (*n* = 3). (**G**) Relative fold change of IRS-1, PGC-1α, and PTP1B in mRNA levels among different groups (*n* = 3). (**H**) Representative Western blot bands for PGC-1α and PTP1B expression in HepG2 cells. (**I**) Quantification results of PGC-1α and PTP1B in HepG2 cells. *n* = 3 for each group. Data were presented as mean ± SEM, * *p* < 0.05, ** *p* < 0.01, *** *p* < 0.001.

An IR-HepG2 cell model was constructed to investigate the therapeutic effect of baicalein on hepatic cells, and the detailed grouping information was outlined in Figure 6A. As shown in Figure 6B,C, HepG2 cells in modeling conditions exhibited a significant decrease in PGC-1α expression and an increase in PTP1B expression, which indicated the successful establishment of an IR-HepG2 cell model. Our preliminary experimental results indicated that baicalein did not affect the cell viability of HepG2 cells under 40 μM concentration, and *Glp1r* siRNA2 exhibited the greatest knockdown efficacy (Figure S1A,B). As depicted in Figure 6D,E, significant glucose uptake decrease was observed in IR-HepG2 cells, and improvement effects were detected in baicalein and liraglutide administration groups, while these effects were attenuated with *Glp1r* knockdown. We observed similar trends in the glucose consumption assay (Figure 6F). Our mechanism investigation revealed that baicalein ameliorates IR of HepG2 cells through upregulating IRS-1 and PGC-1α and downregulating PTP1B in a GLP-1R-dependent manner (Figure 6G–I).

**Figure 7:**
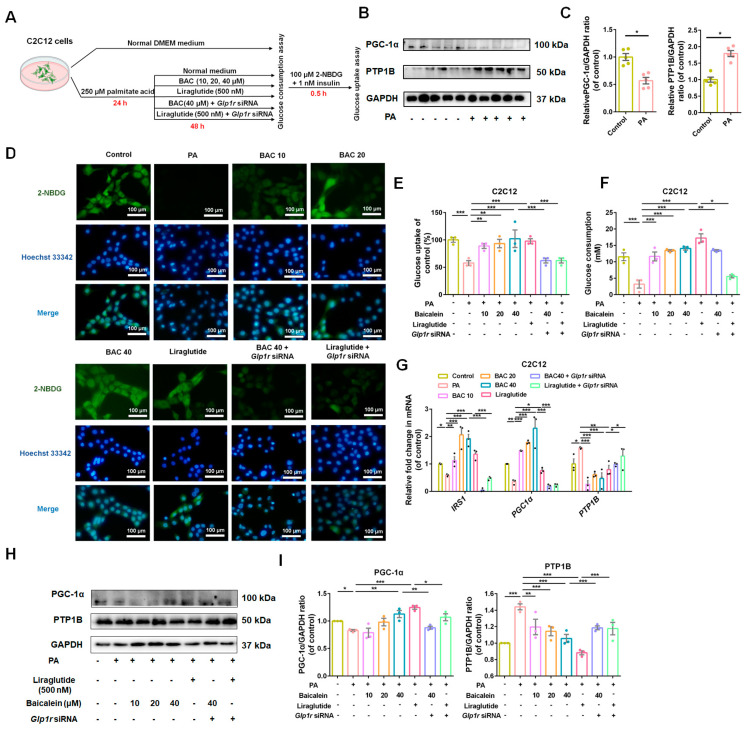
Baicalein improved glucose utilization of insulin resistant (IR) -C2C12 cells. (**A**) Investigation schematic for IR model construction and drug administration in C2C12 cells. (**B**) Representative Western blots bands for PGC-1α and PTP1B in C2C12 cells. (**C**) The quantification results of PGC-1α/GAPDH and PTP1B/GAPDH in C2C12 cells (*n* = 5). (**D**) Immunofluorescence staining of 2-NBDG positive cells in control, PA (250 μM), liraglutide (500 nM), baicalein (10, 20 and 40 μM) combined with *Glp1r* knock down or not. Scale bar: 100 μm. (**E**) Glucose uptake rate determined among different administration groups (*n* = 3). (**F**) The glucose consumption in C2C12 cells given different administrations (*n* = 3). (**G**) Relative fold change of IRS-1, PGC-1α and PTP1B in mRNA among groups given different drug administrations (*n* = 3). (**H**) Representative Western blots bands for PGC-1α and PTP1B expression in C2C12 cells. (**I**) Quantification results of PGC-1α and PTP1B in C2C12 cells. *n*= 3 for each group. Data were presented as mean ± SEM, * *p* < 0.05, ** *p* < 0.01, *** *p* < 0.001.

The IR-C2C12 cell model was also established to simulate the circumstances of skeletal muscle cells under IR conditions (Figure 7A). As shown in Figure 7B,C, a significant decrease in PGC-1α and an evident increase in PTP1B protein level was observed in PA-administrated C2C12 cells. Our preparatory experiments indicated that dosages of baicalein below 40 μM are safe for C2C12 cells, and *Glp1r* siRNA2 is the appropriate siRNA for receptor silence (Figure S1C,D). Baicalein enhanced the glucose uptake and consumption of IR-C2C12 cells in a GLP-1R-dependent manner (Figure 7D–F). Baicalein might act to increase glucose utilization through upregulating PGC-1α and IRS-1, while downregulating PTP1B (Figure 7G–I). 

Taken together, the results acquired in HepG2 and C2C12 indicated that baicalein ameliorates glucose utilization deficits in liver and skeletal muscle tissues under IR condition in a GLP-1R-dependent manner.

**Figure 8:**
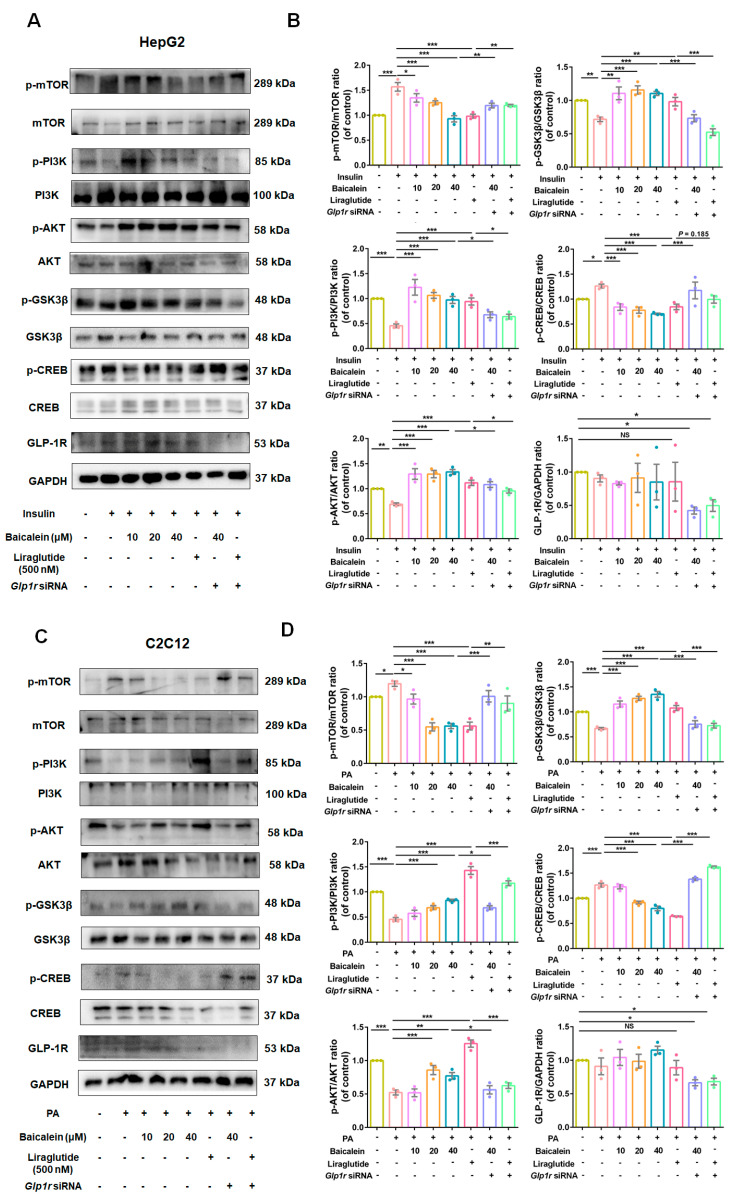
Baicalein improved glucose utilization of IR-HepG2 and IR-C2C12 cells through the PI3K/AKT signaling pathway in a GLP-1R-dependent manner. (**A**) Representative Western blots for p-mTOR, mTOR, p-PI3K, PI3K, p-AKT, AKT, p-GSK3β, GSK3β, p-CREB, CREB, and GLP-1R in HepG2 cells. (**B**) The quantification results of p-mTOR/mTOR, p-PI3K/PI3K, p-AKT/AKT, p-GSK3β/GSK3β, p-CREB/CREB, and GLP-1R/GAPDH ratios in HepG2 cells (*n* = 3). (**C**) Representative Western blots for p-mTOR, mTOR, p-PI3K, PI3K, p-AKT, AKT, p-GSK3β, GSK3β, p-CREB, CREB, and GLP-1R in C2C12 cells. (**D**) The result of quantitative analysis of above proteins in C2C12 cells (*n* = 3). Data were expressed as mean ± SEM, * *p* < 0.05, ** *p* < 0.01, *** *p* < 0.001, one-way ANOVA. NS: no significance.

The protein levels related to the PI3K/AKT insulin signaling pathway were also detected in HepG2 and C2C12 cells, yielding results similar to those observed in vivo pharmacological experiments. As shown in Figure 8A,B, under IR conditions, the phosphorylation levels of mTOR and CREB were increased, while PI3K, AKT, and GSK3β were decreased in HepG2 cells. The administration of baicalein and liraglutide restored these phosphorylation proteins to normal, dependent on the presence of GLP-1R. However, with the knockdown of *Glp1r* the restorative effect was diminished. A similar phenomenon was observed in C2C12 cells (Figure 8C,D).

Collectively, these findings indicated that baicalein mitigates IR through modulating the PI3K/AKT insulin signaling pathway in a GLP-1R-dependent manner.

### 3.6. Baicalein Increased Intracellular Calcium (Ca^2+^) Levels, Activated CaMKII and AMPK, and Led Glucose Transporter 4 (GLUT4) Translocation from Nucleus to Cytomembrane in Skeletal Muscle Cells

**Figure 9:**
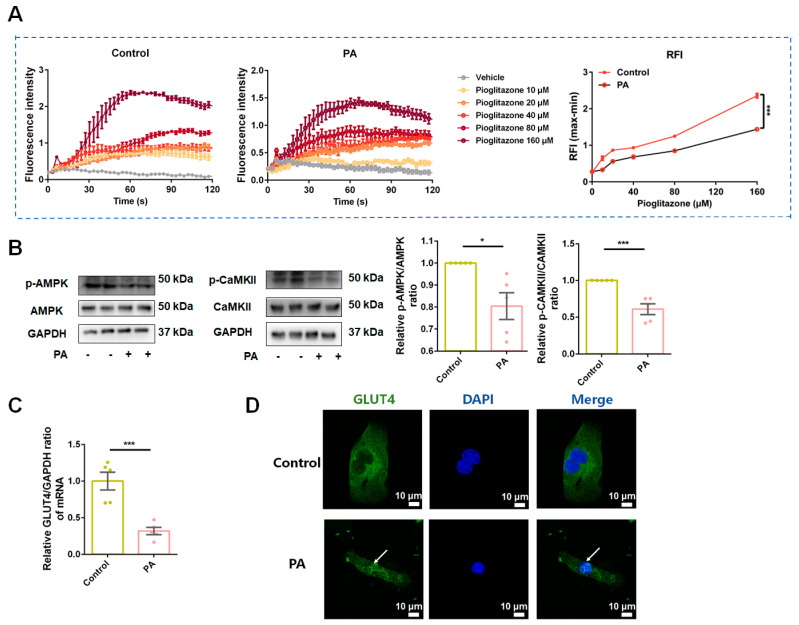
Ca^2+^/CaMKII-AMPK signal pathway impairment and GLUT4 nuclear translocation occurred simultaneously in C2C12 cells under insulin resistance (IR) conditions. (**A**) The effect of pioglitazone administration on intracellular Ca^2+^ level in C2C12 cells. The time-fluorescence intensity change curve of the Ca^2+^ signal in normal conditions (left) and IR conditions (middle). The quantification results of relative fluorescence intensity (RFI) change with pioglitazone administration (right). *n* = 6 for each group. (**B**) Representative Western blot bonds (left) and quantification results of p-AMPK/AMPK and p-CaMKII/CaMKII ratios in C2C12 cells under normal and IR conditions (right). *n* = 5 for each group. (**C**) Quantification results of relative GLUT4/GAPDH mRNA levels in C2C12 cells. *n* = 5 for each group. (**D**) Immunofluorescence staining images of GLUT4 in normal and IR conditions of C2C12 cells. White arrow: GLUT4 nuclear translocation. Scale bar: 10 μm. Data were expressed as mean ± SEM, * *p* < 0.05 *** *p* < 0.001.

Skeletal muscle plays a pivotal role in regulating glucose homeostasis, and is responsible for approximately 80% of postprandial glucose management from the peripheral environment [40]. GLUT4, abundantly expressed in skeletal muscle, plays a crucial role in physical blood glucose utilization and translocates from nuclear to plasma membrane in response to stimuli, including insulin, exercise, and muscle contraction [41]. AMPK is a main downstream effector activated in skeletal muscle in response to these stimuli [42]. Ca^2+^ and calmodulin-dependent protein kinases (CAMKs) are signal factors involved in AMPK signaling pathways [43]. CaMKII, an upstream regulator of AMPK, is phosphorylated during muscle contraction, leading to enhanced glucose uptake [44]. As depicted in Figure 9A, compared to the control condition, the Ca^2+^ flux response of C2C12 cells to pioglitazone, a well-known insulin sensitizer, was discounted under IR conditions. Additionally, a decreased phosphorylation of CaMKII and AMPK (Figure 9B) along with a decrease in GLUT4 mRNA expression (Figure 9C) and plasma membrane translocation (Figure 9D) were observed in IR-C2C12 cells.

**Figure 10:**
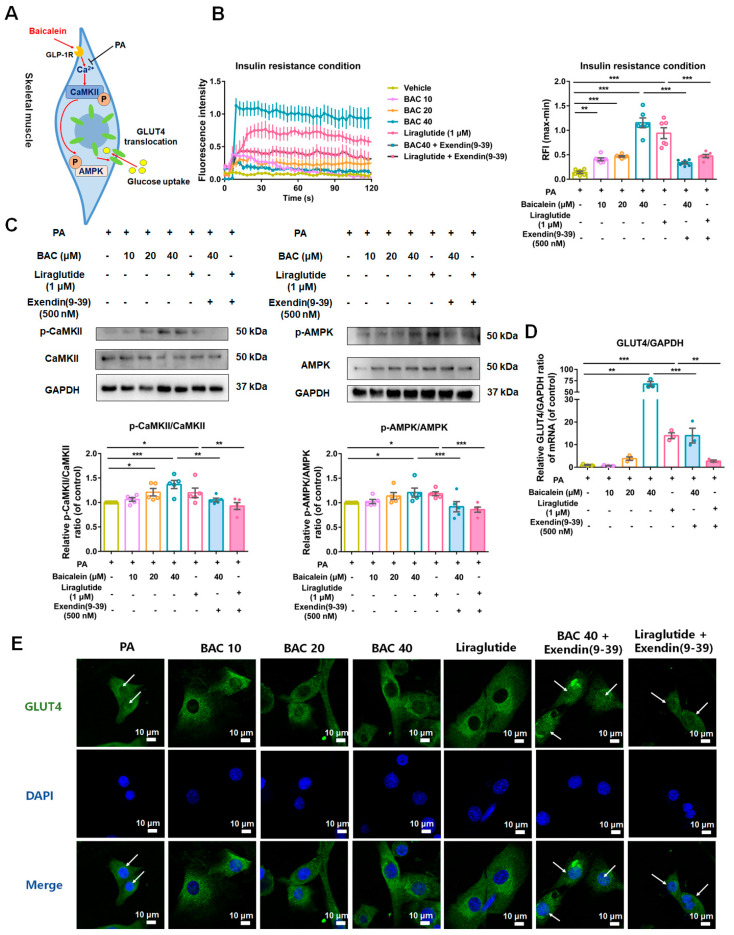
Baicalein enhanced glucose uptake of skeletal muscle through the Ca^2+^/CaMKII-AMPK-GLUT4 signaling pathway in a GLP-1R-dependent manner. (**A**) The schematic sketch of baicalein’s effect on the Ca^2+^/CaMKII-AMPK-GLUT4 signal pathway. (**B**) The intracellular Ca^2+^ flux changes of C2C12 cells given with baicalein (BAC, 10, 20, and 40 μM), liraglutide (1 μM), and exendin (9–39) (500 nM) administration. The time-fluorescence intensity change curve of Ca^2+^ signal in C2C12 cells (left). The quantification results of relative fluorescence intensity (RFI) changes of IR-C2C12 cells given with different administrations (right) (*n* = 6). (**C**) Representative Western blotting bonds and quantification results of p-AMPK/AMPK and p-CaMKII/CaMKII ratios in IR-C2C12 cells given different administrations (*n* = 5). (**D**) Quantification results of GLUT4/GAPDH mRNA levels in IR-C2C12 cells given different administrations (*n* = 3). (**E**) The GLUT4 immunofluorescence staining images of IR-C2C12 cells. White arrow: GLUT4 nuclear translocation. Scale bar: 10 μm. Data were expressed as mean ± SEM. * *p* < 0.05, ** *p* < 0.01, *** *p* < 0.001.

Based on the information obtained above, we hypothesized that GLP-1R might serve as a crucial upstream signal factor in the Ca^2+^/CAMKII-AMPK-GLUT4 signaling pathway (Figure 10A). As shown in Figure 10B, baicalein dose-dependently increased the intracellular Ca^2+^ flux of IR-C2C12 cells, and its effect was attenuated with the inhibition of GLP-1R. Liraglutide, a well-known GLP-1R agonist, and exendin (9–39), a general antagonist of GLP-1R, were applied to verify our hypothesis [9,45]. Our mechanism research indicated that baicalein and liraglutide enhance the phosphorylation of CaMKII and AMPK (Figure 10C), increase the mRNA level of GLUT4 (Figure 10D), and promote the translocation of GLUT4 from the nucleus to the cell membrane (Figure 10E) of IR-C2C12 cells in a GLP-1R-dependent manner.

In conclusion, our findings suggest that baicalein enhances glucose uptake in skeletal muscle cells under IR conditions through the Ca^2+^/CaMKII-AMPK-GLUT4 signaling pathway in a GLP-1R-dependent manner.

## 4. Discussion

DM, one of the fastest-growing diseases worldwide, is projected to affect 693 million adults by 2045 [46]. Oral hypoglycemic agents and exogenous insulin supplementation are the prevailing therapeutic strategies for DM [47]. Therapeutics based on GLP-1R as novel anti-diabetic drugs have been applied in the treatment of diabetes and obesity for decades [48,49]. Increasing evidence has demonstrated that GLP-1R agonists exhibit a favorable benefit/risk profile and reduce the risk of myocardial infarction, stroke, cognitive disorder, and cardiovascular death [50,51]. Presently, the overwhelming majority of GLP-1R agonists applied to clinical therapy are mainly GLP-1 analogues, which are primarily administered via subcutaneous injection [52]. Semaglutide, an existing exception, can be delivered orally but depending on a complex dosing regimen, even so whose oral bioavailability remains below 1% [53]. Nonpeptide agonists, which are designed to directly activate GLP-1R, provide a standardized drug formulation and a simple dosing regimen, offering a promising option for T2DM patients requiring additional daily medications [54]. Historically, great efforts have been dedicated to discovering small molecular agonists that can directly activate GLP-1R. The discovery of LY3502970 [52], RGT1383 [55], compound 2 [56], and TT15 [57] has highlighted the potency of these compounds in GLP-1R activation. Our research has been devoted to discovering natural GLP-1R agonists from traditional Chinese medicine, where baicalein has been preliminary identified as a potential GLP-1R agonist. In this study, we confirmed the hypoglycemic effect of baicalein and further clarified its underlying mechanism. Unexpectedly, the hypoglycemic effect of baicalein on T2DM did not exhibit significant dose dependence, as the FBG levels in WT mice in BAC 50 and BAC 100 groups were lower than those in the BAC 200 group at most time points after drug administration. We attribute this to the limited receptor availability and the sensitivity of β-arrestin recruitment signals, and the subsequent inevitable receptor internalization and desensitization [58,59,60]. GLP-1R agonists have exhibited potent therapeutic effects on IR [4], providing benefits for the treatment of polycystic ovary syndrome [61], neurological dysfunctions [62], NAFLD, and obesity [63]. An impaired mTOR/PI3K/AKT signal pathway is a common pathological feature in these diseases [64]. Our findings revealed that baicalein enhances glucose utilization in liver and skeletal muscle tissues through the PI3K/AKT signaling pathway in a GLP-1R-dependent manner. PTP1B, an important negative regulator of insulin signal transduction pathways, is expressed cumulatively in multiple IR-related diseases [65,66]. PGC-1α, a transcriptional coactivator, is highly expressed in tissues with high energy demands, exhibiting a significant correlation with the pathogenesis of metabolic syndromes such as obesity, T2DM, cardiovascular disease, and hepatic steatosis [67]. We found that the expression of PGC-1α is downregulated and PTP1B is upregulated under IR conditions both in vivo and in vitro, and that baicalein restores these protein levels to normal.

Baicalein, a kind of B-ring unsubstituted flavones, exists widely in multiple natural herbal products. It is of vital importance that research investigates its pharmacokinetic characteristics for further application [68]. Baicalein has excellent absorption properties, exhibited in the t_max_ detected about 5 min after oral administration in mice, as well as excellent permeability in the gut [69,70]. The distribution feature of baicalein is of higher concentrations in the liver and kidneys compared to plasma, similar concentrations in the prostate and plasma, and lower concentrations in the pancreas and lungs [71]. Baicalein is rapidly metabolized in the jejunum and ileum, and then supplementarily biotransformed in the liver, while what remains unabsorbed undergoes further metabolism by bacterial enzymes in the colon [72]. Regarding excretion, the unchanged baicalein in urine accounts for about 0.7%, whereas 27.1% is accounted for in feces [73].

Pioglitazone, a representative thiazolidinedione, is a potent insulin sensitizer with remarkable effects on IR [74,75]. In our mechanism research, pioglitazone was applied as a positive control to validate our hypothesis that the Ca^2+^ flux of skeletal muscle is impaired under IR conditions. As expected, we observed a crippled Ca^2+^/CaMKII-AMPK-GLUT4 signaling pathway in IR-C2C12 cells, while baicalein and liraglutide rescued the abnormal signal pathway in a GLP-1R-dependent manner. All of this indicates that GLP-1R serves as a primary upstream signal factor for the Ca^2+^/CaMKII-AMPK-GLUT4 signal pathway, and this pathway might partly participate in the glucose uptake enhancement effect of baicalein on skeletal muscle.

## 5. Conclusions

**Figure 11:**
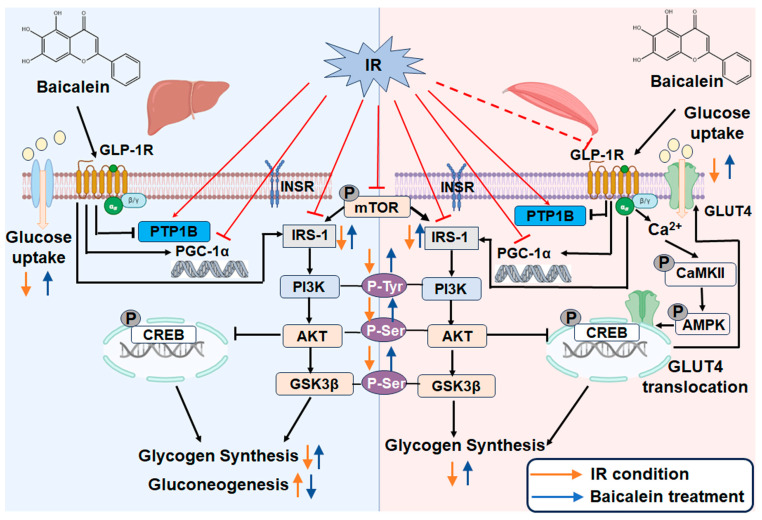
Overview of the therapeutic effect of baicalein against insulin resistance (IR). Under IR conditions, protein tyrosine phosphatase 1B (PTP1B) is overexpressed, phosphorylation levels of mammalian target of rapamycin (mTOR) and cAMP-responsive element–binding protein (CREB) are enhanced, whereas peroxisome proliferator-activated receptor-γ coactivator (PGC)-1α and PI3K/AKT insulin pathways are inactive both in liver and skeletal muscle tissues. Baicalein improves glucose metabolism via enhancing phosphorylation of the PI3K/AKT/glycogen synthase kinase-3β (GSK-3β) signal pathway, increasing dephosphorylation of mTOR and CREB, resulting in an increase in glycogen synthesis and a decrease in gluconeogenesis both in liver and skeletal muscle. Baicalein also improves glucose uptake by skeletal muscles through improving Ca^2+^/calmodulin-dependent protein kinases II (CaMKII)-5′-adenosine monophosphate-activated protein kinase (AMPK) turbulence and reversing glucose transporter 4 (GLUT4) nuclear translocation in a GLP-1R-dependent manner. →  (Direct stimulatory modification); ┴ (Direct Inhibitory modification); ↑ (Up regulate); ↓ (Down regulate); PI3K (Phosphoinositide 3-Kinase); AKT (protein kinase B); GLP-1R (Glucagon-like peptide-1 receptor); INSR (insulin receptor); IRS-1 (insulin Receptor Substrate-1).

In conclusion, our results indicate that baicalein improves the glucose metabolism of diabetes through ameliorating IR occurring in liver and skeletal muscle tissues with the existence of GLP-1R. The detailed mechanism is summarized in Figure 11.

## Figures and Tables

**Table 1:** Primer sequences used for RT-PCR.

	Accession Number	Forward	Reverse	Product Length
IRS-1	NM_005544.3	ACTGGACATCACAGCAGAATGA	AGAACGTGCAGTTCAGTCAA	199
PTP1B	NM_011201.3	TGACGGTGCTCATGACTCTT	TGCTGGCTTCTCTGGGTAAA	141
GLUT4	NM_001359114.2	CAACTGGACCTGTAACTTCATTGT	ACGGCAAATAGAAGGAAGACGTA	87
PGC-1α	NM_001402988.1	CACCAAACCCACAGAAAACAG	GGGTCAGAGGAAGAGATAAAGTTG	125
GAPDH	NM_002046.7	GAGTCAACGGATTTGGTCGT	GACAAGCTTCCCGTTCTCAG	185

## Data Availability

All data were generated in-house, and no paper mill was used. All authors agreed to be accountable for all aspects of the work ensuring integrity and accuracy. The raw data supporting the conclusions of this article will be made available by the authors on request.

## Supplementary Materials

The following supporting information can be downloaded at: https://www.mdpi.com/article/10.3390/antiox13101246/s1, Table S1. The pathways acquired by KEGG enrichment analysis. Figure S1. The influence of baicalein and liraglutide’s administration on cell viability of HepG2 and C2C12 cells, and the determination for optimal siRNA for *Glp1r* silence. (A) The cell viability of HepG2 cells given with different concentration of baicalein and liraglutide (*n* = 5). (B) Representative Western blotting bands (left) and quantification results (right, *n* = 3) of HepG2 cells administrated with different siRNAs. (C) The cell viability of C2C12 cells given with different concentrations of baicalein and liraglutide (*n* = 5). (D) Representative Western blotting bands (left) and quantification results (right, *n* = 3) of C2C12 cells administrated with different siRNAs. Data were presented as mean ± SEM, * *p* < 0.05, ** *p* < 0.01, *** *p* < 0.001.
