# Supplementary material for: Baicalein Ameliorates Insulin Resistance of HFD/STZ Mice Through Activating PI3K/AKT Signal Pathway of Liver and Skeletal Muscle in a GLP-1R-Dependent Manner
Source: Antioxidants (Basel). 2024 Oct 16;13(10):1246. doi: 10.3390/antiox13101246 (PMC11505556; doi:10.3390/antiox13101246)
Supplement: Supplementary file 1 [file antioxidants-13-01246-s001.zip › antioxidants-3215240-supplementary.pdf]

Table S1 The pathways acquired by KEGG enrichment analysis.

Term	Count	%	P Value
hsa05200: Pathways in cancer	52	45.61404	3.01E-33
hsa05417: Lipid and atherosclerosis	34	29.82456	1.95E-27
hsa05167: Kaposi sarcoma-associated herpesvirus infection	33	28.94737	1.30E-27
hsa04151: PI3K-Akt signaling pathway	33	28.94737	3.60E-19
hsa05163: Human cytomegalovirus infection	31	27.19298	4.84E-23
hsa05022: Pathways of neurodegeneration - multiple diseases	30	26.31579	5.86E-13
hsa05010: Alzheimer disease	28	24.5614	1.43E-13
hsa05161: Hepatitis B	27	23.68421	4.39E-22
hsa05205: Proteoglycans in cancer	25	21.92982	3.99E-17
hsa04010: MAPK signaling pathway	25	21.92982	2.69E-13
hsa04933: AGE-RAGE signaling pathway in diabetic complications	24	21.05263	2.33E-23
hsa05162: Measles	24	21.05263	5.82E-20
hsa05418: Fluid shear stress and atherosclerosis	24	21.05263	6.90E-20
hsa05131: Shigellosis	24	21.05263	3.32E-14
hsa05132: Salmonella infection	24	21.05263	3.95E-14
hsa05165: Human papillomavirus infection	24	21.05263	1.59E-11
hsa05160: Hepatitis C	23	20.17544	2.24E-17
hsa05164: Influenza A	23	20.17544	1.26E-16
hsa05169: Epstein-Barr virus infection	23	20.17544	4.90E-15
hsa05208: Chemical carcinogenesis - reactive oxygen species	23	20.17544	3.89E-14
hsa05215: Prostate cancer	22	19.29825	8.90E-21
hsa01522: Endocrine resistance	22	19.29825	1.12E-20
hsa05152: Tuberculosis	22	19.29825	5.43E-15
hsa05212: Pancreatic cancer	21	18.42105	1.14E-21
hsa04657: IL-17 signaling pathway	21	18.42105	1.17E-19
hsa04066: HIF-1 signaling pathway	21	18.42105	2.66E-18
hsa05145: Toxoplasmosis	21	18.42105	3.89E-18
hsa05135: Yersinia infection	21	18.42105	2.86E-16
hsa04932: Non-alcoholic fatty liver disease	21	18.42105	3.55E-15
hsa05225: Hepatocellular carcinoma	21	18.42105	1.74E-14
hsa05207: Chemical carcinogenesis-receptor activation	21	18.42105	1.51E-12
hsa05170: Human immunodeficiency virus 1 infection	21	18.42105	1.51E-12
hsa05166: Human T-cell leukemia virus 1 infection	21	18.42105	3.58E-12
hsa05206: MicroRNAs in cancer	21	18.42105	1.50E-09
hsa04659: Th17 cell differentiation	20	17.54386	4.60E-17
hsa04068: FoxO signaling pathway	20	17.54386	2.04E-15
hsa04936: Alcoholic liver disease	20	17.54386	9.52E-15
hsa05224: Breast cancer	20	17.54386	1.82E-14
hsa05130: Pathogenic Escherichia coli infection	20	17.54386	4.29E-12
hsa04014: Ras signaling pathway	20	17.54386	9.49E-11
hsa05168: Herpes simplex virus 1 infection	20	17.54386	2.03E-05
hsa05235: PD-L1 expression and PD-1 checkpoint pathway in cancer	19	16.66667	2.39E-17
hsa05142: Chagas disease	19	16.66667	3.11E-16
hsa04668: TNF signaling pathway	19	16.66667	2.53E-15
hsa04210: Apoptosis	19	16.66667	6.22E-14
hsa04621: NOD-like receptor signaling pathway	19	16.66667	1.45E-11

hsa05171: Coronavirus disease - COVID-19	19	16.66667	5.74E-10
hsa05210: Colorectal cancer	18	15.78947	2.97E-16
hsa05222: Small cell lung cancer	18	15.78947	9.84E-16
hsa04625: C-type lectin receptor signaling pathway	18	15.78947	8.86E-15
hsa05226: Gastric cancer	18	15.78947	3.90E-12
hsa04015: Rap1 signaling pathway	18	15.78947	9.43E-10
hsa04917: Prolactin signaling pathway	17	14.91228	1.99E-16
hsa05133: Pertussis	17	14.91228	8.15E-16
hsa01521: EGFR tyrosine kinase inhibitor resistance	17	14.91228	1.68E-15
hsa04620: Toll-like receptor signaling pathway	17	14.91228	2.86E-13
hsa04660: T cell receptor signaling pathway	17	14.91228	1.76E-12
hsa04931: Insulin resistance	16	14.03509	4.42E-12
hsa04218: Cellular senescence	16	14.03509	9.21E-10
hsa05223: Non-small cell lung cancer	15	13.15789	1.95E-13
hsa01524: Platinum drug resistance	15	13.15789	2.39E-13
hsa05140: Leishmaniasis	15	13.15789	5.20E-13
hsa04211: Longevity regulating pathway	15	13.15789	4.17E-12
hsa04919: Thyroid hormone signaling pathway	15	13.15789	2.96E-10
hsa04926: Relaxin signaling pathway	15	13.15789	7.02E-10
hsa05202: Transcriptional misregulation in cancer	15	13.15789	1.29E-07
hsa04510: Focal adhesion	15	13.15789	2.42E-07
hsa05203: Viral carcinogenesis	15	13.15789	2.57E-07
hsa05020: Prion disease	15	13.15789	7.93E-06
hsa05219: Bladder cancer	14	12.2807	1.37E-15
hsa05221: Acute myeloid leukemia	14	12.2807	1.54E-12
hsa05218: Melanoma	14	12.2807	4.08E-12
hsa05220: Chronic myeloid leukemia	14	12.2807	8.42E-12
hsa04064: NF-kappa B signaling pathway	14	12.2807	5.03E-10
hsa04722: Neurotrophin signaling pathway	14	12.2807	2.74E-09
hsa04071: Sphingolipid signaling pathway	14	12.2807	3.37E-09
hsa04380: Osteoclast differentiation	14	12.2807	1.30E-08
hsa04915: Estrogen signaling pathway	14	12.2807	1.55E-08
hsa04062: Chemokine signaling pathway	14	12.2807	8.32E-07
hsa05213: Endometrial cancer	13	11.40351	5.18E-12
hsa05214: Glioma	13	11.40351	1.27E-10
hsa05146: Amoebiasis	13	11.40351	4.94E-09
hsa04140: Autophagy - animal	13	11.40351	1.06E-06
hsa04630: JAK-STAT signaling pathway	13	11.40351	1.13E-06
hsa05415: Diabetic cardiomyopathy	13	11.40351	9.24E-06
hsa05014: Amyotrophic lateral sclerosis	13	11.40351	0.002157
hsa05134: Legionellosis	12	10.52632	7.64E-11
hsa04658: Th1 and Th2 cell differentiation	12	10.52632	1.89E-08
hsa04152: AMPK signaling pathway	12	10.52632	3.30E-07
hsa04910: Insulin signaling pathway	12	10.52632	1.16E-06
hsa04150: mTOR signaling pathway	12	10.52632	4.15E-06
hsa04217: Necroptosis	12	10.52632	5.00E-06
hsa04613: Neutrophil extracellular trap formation	12	10.52632	2.84E-05
hsa04024: cAMP signaling pathway	12	10.52632	1.26E-04
hsa05012: Parkinson disease	12	10.52632	5.35E-04

hsa05016: Huntington disease	12	10.52632	0.001683
hsa04213: Longevity regulating pathway - multiple species	11	9.649123	3.76E-09
hsa05321: Inflammatory bowel disease	11	9.649123	7.16E-09
hsa05230: Central carbon metabolism in cancer	11	9.649123	1.50E-08
hsa04115: p53 signaling pathway	11	9.649123	2.61E-08
hsa04520: Adherens junction	11	9.649123	2.40E-07
hsa04935: Growth hormone synthesis, secretion and action	11	9.649123	2.60E-06
hsa04072: Phospholipase D signaling pathway	11	9.649123	1.69E-05
hsa04370: VEGF signaling pathway	10	8.77193	4.54E-08
hsa04920: Adipocytokine signaling pathway	10	8.77193	1.84E-07
hsa05120: Epithelial cell signaling in Helicobacter pylori infection	10	8.77193	2.09E-07
hsa04012: ErbB signaling pathway	10	8.77193	1.13E-06
hsa05323: Rheumatoid arthritis	10	8.77193	2.43E-06
hsa04921: Oxytocin signaling pathway	10	8.77193	1.41E-04
hsa04934: Cushing syndrome	10	8.77193	1.48E-04
hsa01523: Antifolate resistance	9	7.894737	2.42E-09
hsa05216: Thyroid cancer	9	7.894737	1.48E-08
hsa04930: Type II diabetes mellitus	9	7.894737	9.11E-08
hsa05144: Malaria	9	7.894737	1.80E-07
hsa04662: B cell receptor signaling pathway	9	7.894737	1.02E-05
hsa05231: Choline metabolism in cancer	9	7.894737	3.18E-05
hsa04914: Progesterone-mediated oocyte maturation	9	7.894737	4.25E-05
hsa04371: Apelin signaling pathway	9	7.894737	3.70E-04
hsa04550: Signaling pathways regulating pluripotency of stem cells	9	7.894737	4.48E-04
hsa04060: Cytokine-cytokine receptor interaction	9	7.894737	0.034858
hsa04215: Apoptosis - multiple species	8	7.017544	1.13E-07
hsa04664: Fc epsilon RI signaling pathway	8	7.017544	2.24E-05
hsa05211: Renal cell carcinoma	8	7.017544	2.47E-05
hsa04622: RIG-I-like receptor signaling pathway	8	7.017544	2.98E-05
hsa04623: Cytosolic DNA-sensing pathway	8	7.017544	4.27E-05
hsa04640: Hematopoietic cell lineage	8	7.017544	2.50E-04
hsa04725: Cholinergic synapse	8	7.017544	5.60E-04
hsa04810: Regulation of actin cytoskeleton	8	7.017544	0.026283
hsa04137: Mitophagy - animal	7	6.140351	2.89E-04
hsa04912: GnRH signaling pathway	7	6.140351	0.001136
hsa04670: Leukocyte transendothelial migration	7	6.140351	0.00335
hsa04611: Platelet activation	7	6.140351	0.004851
hsa04022: cGMP-PKG signaling pathway	7	6.140351	0.019343
hsa04310: Wnt signaling pathway	7	6.140351	0.023148
hsa04360: Axon guidance	7	6.140351	0.028081
hsa04913: Ovarian steroidogenesis	6	5.263158	4.44E-04
hsa04923: Regulation of lipolysis in adipocytes	6	5.263158	8.07E-04
hsa05416: Viral myocarditis	6	5.263158	9.43E-04
hsa04750: Inflammatory mediator regulation of TRP channels	6	5.263158	0.008022
hsa04928: Parathyroid hormone synthesis, secretion and action	6	5.263158	0.011057
hsa04650: Natural killer cell mediated cytotoxicity	6	5.263158	0.02254
hsa04114: Oocyte meiosis	6	5.263158	0.025366
hsa04261: Adrenergic signaling in cardiomyocytes	6	5.263158	0.045983
hsa04110: Cell cycle	6	5.263158	0.04924

hsa00220: Arginine biosynthesis	5	4.385965	1.51E-04
hsa05143: African trypanosomiasis	5	4.385965	0.001176
hsa04960: Aldosterone-regulated sodium reabsorption	5	4.385965	0.001176
hsa02010: ABC transporters	5	4.385965	0.002454
hsa05217: Basal cell carcinoma	5	4.385965	0.008243
hsa04929: GnRH secretion	5	4.385965	0.008709
hsa05100: Bacterial invasion of epithelial cells	5	4.385965	0.016371
hsa04146: Peroxisome	5	4.385965	0.020989
hsa04976: Bile secretion	5	4.385965	0.026336
hsa04061: Viral protein interaction with cytokine and cytokine receptor	5	4.385965	0.038093
hsa04916: Melanogenesis	5	4.385965	0.03929
hsa05030: Cocaine addiction	4	3.508772	0.024034
hsa00330: Arginine and proline metabolism	4	3.508772	0.025337

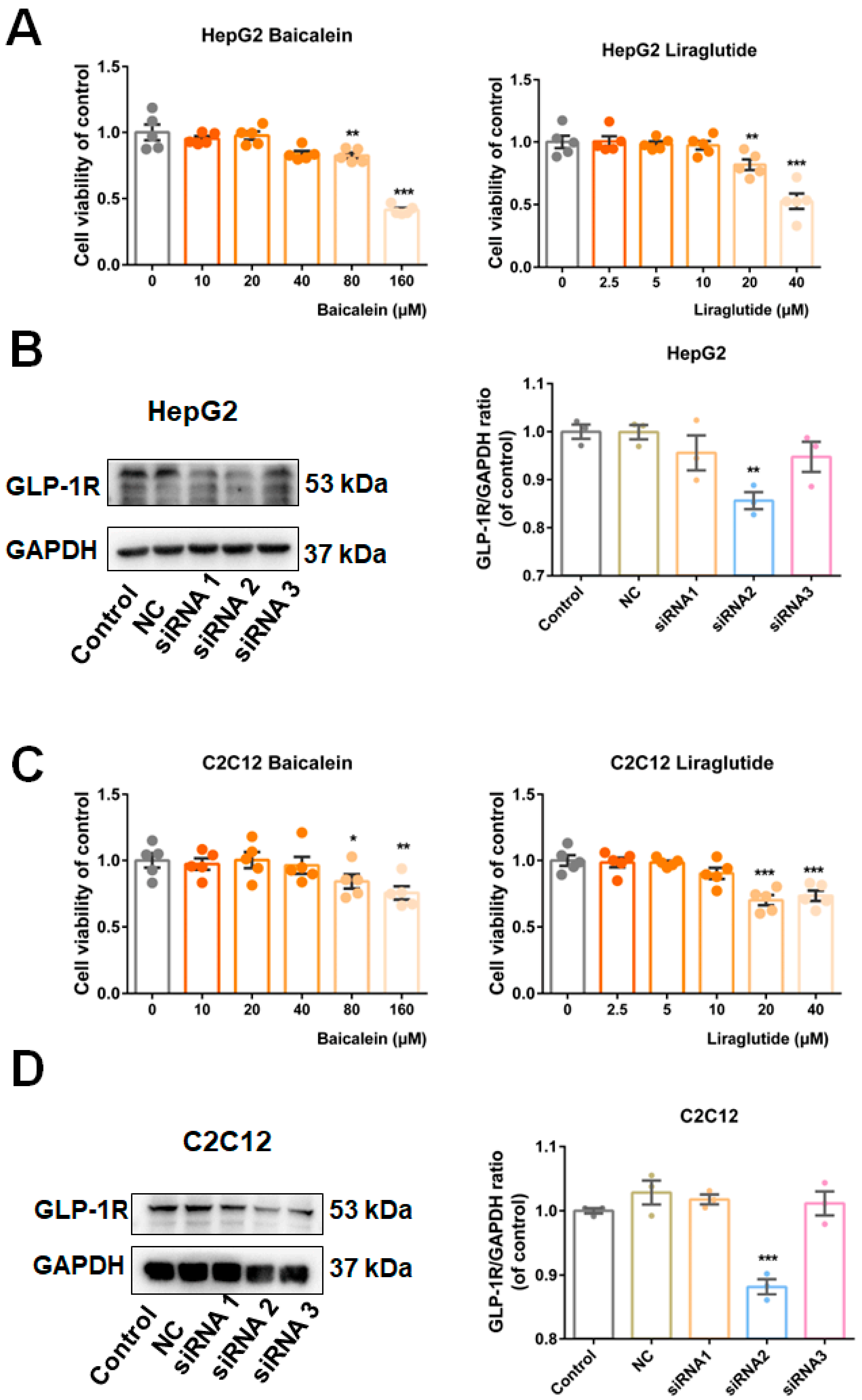

Figure S1. The influence of baicalein and liraglutide's administration on cell viability of HepG2 and C2C12 cells, and the determination for optimal siRNA for *Glp1r* silence. (A) The cell viability of HepG2 cells given with different concentrations of baicalein and liraglutide ($n = 5$). (B) Representative Western blotting bands (left) and quantification results (right, $n = 3$) of HepG2 cells transfected with different siRNAs. (C)

The cell viability of C2C12 cells given with different concentrations of baicalein and liraglutide ($n = 5$). (D) Representative Western blotting bands (left) and quantification results (right, $n = 3$) of C2C12 cells administrated with different siRNAs. Data were presented as mean ± SEM, $^{*}P < 0.05$, $^{**}P < 0.01$, $^{***}P < 0.001$.
